# Rapid Magneto-Sonoporation of Adipose-Derived Cells

**DOI:** 10.3390/ma14174877

**Published:** 2021-08-27

**Authors:** Miriam Filippi, Boris Dasen, Arnaud Scherberich

**Affiliations:** 1 Soft Robotics Laboratory, ETH Zurich, Tannenstrasse 3, 8092 Zurich, Switzerland; 2 Department of Biomedicine, University Hospital Basel, University of Basel, Hebelstrasse 20, 4031 Basel, Switzerland; boris.dasen@usb.ch; 3 Department of Biomedical Engineering, University of Basel, Gewerbestrasse 14, 4123 Allschwil, Switzerland

**Keywords:** magneto-sonoporation, magnetic nanoparticles, stem cells, superparamagnetic iron oxide particles, cell labelling, ultrasounds, osteogenesis, bone tissue engineering

## Abstract

By permeabilizing the cell membrane with ultrasound and facilitating the uptake of iron oxide nanoparticles, the magneto-sonoporation (MSP) technique can be used to instantaneously label transplantable cells (like stem cells) to be visualized via magnetic resonance imaging in vivo. However, the effects of MSP on cells are still largely unexplored. Here, we applied MSP to the widely applicable adipose-derived stem cells (ASCs) for the first time and investigated its effects on the biology of those cells. Upon optimization, MSP allowed us to achieve a consistent nanoparticle uptake (in the range of 10 pg/cell) and a complete membrane resealing in few minutes. Surprisingly, this treatment altered the metabolic activity of cells and induced their differentiation towards an osteoblastic profile, as demonstrated by an increased expression of osteogenic genes and morphological changes. Histological evidence of osteogenic tissue development was collected also in 3D hydrogel constructs. These results point to a novel role of MSP in remote biophysical stimulation of cells with focus application in bone tissue repair.

## 1. Introduction

Due to their responsiveness to externally applied magnetic fields, magnetic nanoparticles (MNPs) are extremely attractive materials used in a number of biomedical applications [1]. Iron oxide-based nanoparticles (IONPs) are composed of cores of magnetite (Fe_3_O_4_) and its oxidized form maghemite (γ–Fe_2_O_3_), further surrounded by external coatings that increase the stability and biocompatibility of the suspension. The IONPs have been extensively exploited as contrast agents for magnetic resonance imaging (MRI), but thanks to their highly versatile superparamagnetic nature, they have become relevant also in biosensing, gene and drug delivery, engineering of biological tissues, and theranostics, a discipline that combines treatment and diagnosis of various pathologies [2]. The labeling of cells via IONPs enables not only their visualization and real-time monitoring, but also their magnetization. Magnetization of cells allows for the remote control of their functions and spatial localization. As such, this technique has become of great interest for the delivery of therapeutic cells (cell therapy) and manufacturing of transplantable tissue (tissue engineering) [3]. Indeed, magnetizing the cells with multidirectional differentiation potential and self-renewal capabilities, such as stem cells, holds potential in mediating the structural repair and functional recovery of biological tissues. In fact, the IONPs can be used to stimulate the signaling pathways and regulate the cell functions involved in regeneration (magnetic actuation), as well as to spatially guide the cells through magnetic forces (magnetic targeting and printing) [4,5,6,7].

However, regenerative and stem cells are non-phagocytic cells. As such, conventional labeling procedures require long times of incubation and lead to moderate uptake efficiency. Cytotoxicity often occurs at high dosages and long exposure times [8,9,10,11,12]. To enhance the IONP internalization and improve stem cell magnetization, methodological optimization has been widely tested. For instance, since subjecting the cells to physical forces can assist the delivery of bioactive molecules, various techniques based on biophysical cell stimulation have been proposed, including: microinjection, electroporation, laser irradiation, magnetofection, and electric field-induced molecular vibration [13,14,15,16]. However, issues of cytotoxicity are still present.

Mechanical waves, such as ultrasound (US), transiently enhance the permeability of biological membranes (like endothelial layers and cell membranes) and thus facilitate the passage of drugs based on small molecules, plasmidic DNA, and also nanomaterials across layers and barriers. The use of US waves to temporarily permeabilize the cell membrane and elicit the intracellular uptake of exogenous compounds is known as sonoporation, and can be achieved by applying sonication at low frequency (kilohertz), lithotripter shockwaves, high-frequency ultrasound (HIFU), or even diagnostic ultrasound (megahertz frequencies) [17,18,19]. Sonication allows for safe, effective drug and gene delivery, which can be further enhanced by ultrasonically activated microbubbles (MBs) that pulsate in the close proximity of the cells and augment their membrane permeability [18,19,20,21]. Simple and inexpensive, sonoporation also offers the following advantages: minimal requirement in terms of instrumentation; temporal and spatial specificity based on the site of insonation; low risk of immuno-pathogenesis; existing extensive characterization of the approach in vitro and in mammalians; need for moderate energy transfer and easy implementation into clinical approaches; as well as rapid kinetics comparable to those of electroporation (i.e., instant labeling) [18,19]. Moreover, whereas current physical methods allow for instant labeling of limited number of cells, large quantities of cells can be labeled by sonoporation [22,23].

Due to these advantages, sonoporation has emerged among physical cell labeling methods, and it has been extensively used to deliver pharmaceuticals and genetic material [17,19]. During the transient perforation of the membrane, bioactive compounds enter the cells with tunable kinetics which are strongly determined by both acoustic driving parameters and MB-to-cell relative parameters [24,25,26]. Interestingly, in order to enhance the internalization of biochemicals, sonoporation has been applied also in combination with nanomaterials [27]. Liposomes, polymeric nanoparticles, micelles and nanoemulsions enhanced the gene transfection and drug loading efficiency by acting as nanocarriers, stimuli-responsive delivery systems, or co-adjuvant agents [27,28,29]. Some of these works demonstrated that nanosized materials could cross the cell membrane during its permeabilization. However, the use of sonoporation to increase the internalization of IONPs (magneto-sonoporation, MSP) has been tested through only few studies [22,23,30,31,32], which were carried out on progenitor cells with circumscribed regenerative potential and applicability, like neural stem cells and osteosarcoma cell lines [22,23,32].

The adipose-derived stem cells (ASCs) display low immunogenicity and can differentiate into multiple cell types [33]. Furthermore, they can be easily harvested with limited morbidity and then rapidly expanded. As such, they have already been involved in a vast plethora of therapeutic applications at both the pre-clinical and clinical levels. Importantly, ASCs stimulate tissue repair by releasing growth factors [34,35] and undergoing differentiation into muscle, endothelial, bone, cartilage, adipose and nerve tissue [36,37,38,39]. Due to their versatility and potency, these cells hold promise for developing effective cellular therapies in various types of damaged tissues [40,41,42]. Intriguingly, they respond to physical stimuli by activating specific signaling pathways (such as those involved in the differentiation) [43,44], which allows for remote control of their functions [45].

Thereby, the quick and efficient magnetization of ASCs would substantially improve their traceability by MRI and their use in regenerative medicine. In order to enhance the internalization of IONPs and achieve rapid cell magnetization, we thus treated human ASCs with MSP and then characterized the effects of this procedure on the IONP uptake, viability, metabolic activity and differentiation of those cells.

## 2. Materials and Methods

### 2.1. Adipose-Derived Stem Cells (ASC) Culture

The ASCs were isolated from the stromal vascular fraction (SVF) cells extracted from the adipose tissue, as previously reported [46,47]. Liposuctions were obtained from six healthy donors after informed consent and according to a protocol approved by the Ethical Committee of the Canton of Basel (Ethikkommission beider Basel [EKKB], Ref. 78/07). The human adipose tissue biopsies were enzymatically digested (37 °C, 45 min) with 0.075% collagenase type II (Worthington Biochemical Corp., Lakewood, NJ, USA) and centrifuged (1700 rpm, 10 min). Finally, a complete medium (CM) consisting of alpha-modified Eagle’s medium (α-MEM) supplemented with 10% fetal bovine serum (FBS) (Sigma-Aldrich, Schaffausen, Switzerland), 1% HEPES (Sigma-Aldrich, Schaffausen, Switzerland), 1% sodium pyruvate(Sigma-Aldrich, Schaffausen, Switzerland), and 1% penicillin (10,000 U/mL) (Sigma-Aldrich, Schaffausen, Switzerland), streptomycin (10,000 µg/mL) (Sigma-Aldrich, Schaffausen, Switzerland) and L-glutamine (29.2 mg/mL) solution (PSG solution, Thermo Fischer Scientific, Waltham, MA, USA) was used to suspend the cells before they were filtered through a 100 µm strainer (BD Biosciences, Eysins, Vaud, Switzerland) and counted. SVF cells were plated and then, the ASCs were isolated by adherence to plastic during the culture into CM. Cells were incubated in 5% CO_2_ at 37 °C and maintained at sub-confluent levels onto poly-d-lysine (Sigma-Aldrich, Schaffausen, Switzerland)-coated 75 cm^2^ flasks, with medium changes every 72 h. When flasks were confluent, cells were detached with trypsin-EDTA (Thermo Fischer Scientific, Waltham, MA, USA), split and re-plated.

### 2.2. Magneto-Sonoporation

To perform sonoporation, cells were suspended in phosphate-buffered saline (PBS) at a density of approximately 1.5 × 10^6^ cells/mL and mixed with PEG-functionalized iron oxide (II,III) nanoparticles with an average particle size of 15 nm (1 mg/mL in aqueous suspension) purchased from Sigma Aldrich, Schaffausen, Switzerland. MBs of 2.3–2.9 µm of diameter (membrane composition: polyethylene-glycol, phospholipids, and fatty acids; gas mixture: perfluorobutane and nitrogen) used as untargeted US contrast agents (SonoVue) were purchased from BRACCO Research SA (Geneva, Switzerland). After adding the MBs, the plastic tubes containing the mixture were then readily transferred to a custom cup-like container for sonication, which was installed on the top of an ultrasonic transducer connected to an ultrasonic liquid processor sonicator (S4000, Misonix Inc., Farmingdale, NY, USA). To optimize the procedure, the crucial parameters were considered: ultrasonic intensity, duty cycle, and exposure time, which could be adjusted through the digital panel of the ultrasound generator. Water served as transmission medium between the acoustic probe and the specimens, with acoustic powers being tuned as 0.5–2.0 W/cm^2^. First, viability of ASCs alone was evaluated by Trypan Blue exclusion test, upon variable power intensities (0.5, 1.0, 2.0 W/cm^2^), exposure times (5, 10, 30, 60, 120, 300 s), and duty cycles (20%, 50%). Those parameters that elicited high cell viability (>90%) were selected as the sub-optimal parameters for optimizing the procedure in the presence of IONPs (50 µg/mL). The second round of optimization was conducted by comparing the cell viability among various groups by Student’s *t*-test in order to select the setting providing the highest viability and intracellular uptake of iron. The following parameters were selected: 20% duty cycle, 1 W/cm^2^ and 30 s.

### 2.3. Iron Content

The amount of iron in the supernatants of gel cultures was determined by assessing the spectrophotometric properties of SPIO using a 96-well multiplate reader and a standard protocol to obtain spectra from 200–750 nm. The iron was quantified by absorbance at 370 nm, i.e., the one of oxybridged iron (i.e., Fe–O–Fe), found in the SPIO crystal core [48,49]. The spectral analysis of 1× PBS was used as a blank for signal normalization. In this study, the limit of quantification for Fe was 0.20 μg/mL.

### 2.4. Stability of Nanoparticles

In order to assess the overall stability of MNPs in biological media and the effects possibly caused by the US exposure, MNPs were incubated in cell culture medium (dilution ratio, 1:50) and maintained in cell culture conditions (37 °C, 5% CO_2_, pH 7.4) for two days. Dynamic light scattering (DLS, Zetasizer Nano 90 ZS, Malvern, UK) allowed for the determination of the mean hydrodynamic diameter of particles and their polydispersity index (PDI). During the monitoring time, the level of NP aggregation was evaluated in terms of size and PDI changes. In order to calculate the particle size, a scattering angle of 90 degrees was used. All measurements were performed in triplicate at 25 °C.

### 2.5. Prussian Blue Staining of Cells

Following IONP incubation or MSP treatment, the cells were washed twice with PBS and fixed with 4% paraformaldehyde solution (30 min at room temperature). After washing again twice with PBS, cells were exposed to the Prussian blue staining solution, consisting of a 1:1 mixture of 4% hydrochloric acid (Sigma-Aldrich, Schaffausen, Switzerland) and 4% potassium ferrocyanide (Sigma-Aldrich, Schaffausen, Switzerland) for 15 min at room temperature. Cells were washed with distilled water three times, before the counterstaining for cytoplasm with eosin (Panreac Química S.L.U, Barcelona, Spain) for 5 min at room temperature. After washing, the cells were observed using inverted light microscopy (Olympus IX83, Life Science Solutions, Hamburg, Germany). All experiments were carried out in triplicate.

### 2.6. Cell Viability and Proliferation

In order to determine whether the magneto-sonoporation would adversely affect the functions of the ASCs, cell viability and proliferation (metabolic assimilation rate) were evaluated. Cell viability was determined using Trypan Blue exclusion, with subsequent cell counting using a hemocytometer.

### 2.7. Morphology Analysis

Light microscopy and fluorescent phalloidin staining of cells were undertaken at the experimental endpoint. Briefly, 5000 cells per well were plated in order to better visualize individual cell morphology. At the endpoint, cells were washed with phosphate buffer saline (PBS) solution and fixed with 4% (*w/v*) paraformaldehyde for 15 min at room temperature. Following permeabilization with 0.2% (*v/v*) Triton X-100 (Sigma-Aldrich, Schaffausen, Switzerland) for 20 min, cells were stained for 20 min in the dark with Alexa 488-conjugated phalloidin (1:40, Life Technologies, Carlsbad, CA, USA). After PBS washes, cells were imaged using an Olympus IX51 fluorescent microscope at 40× magnification. Fifteen images of cells in each experimental condition were taken, and aspect ratio (AR) for each cell (longest cell length/narrowest cell width) was determined using Image J (National Institutes of Health, Bethesda, MD, USA).

### 2.8. Evaluation of the Membrane Resealing Time

The Methylene Blue dye (Sigma-Aldrich, Schaffausen, Switzerland) (2.5 mM, for 5 min) was added to cells at different time points after the US application. Stained cells were considered as owing a permeable membrane due to sonoporation, and their percentage on the total population was reported.

### 2.9. Gene Expression Analysis

Total RNA was extracted with RNeasy^®^ Mini kit protocol (#74104, Qiagen, Hilden, Germany). All RNAs were treated by Deoxyribonuclease I (DNAse I; Invitrogen, Waltham, MA, USA) and total RNA was reverse-transcribed into cDNA with the Omniscript Reverse Transcription kit (#205111, Qiagen, Hilden, Germany) at 37 °C for 60 min. Quantitative real-time PCR assays were performed with ABIPrism 77000 Sequence Detection System (Perkin Elmer, Schwerzenbach, Switzerland) and utilizing Taqman Universal PCR Master Mix (#4304437, Applied Biosystems, Waltham, MA, USA). The cycling parameters were: 50 °C for 2 min, followed by 95 °C for 10 min and 40 cycles of denaturation at 95 °C for 15 s and annealing/extension at 60 °C for 1 min. Reactions were performed in triplicate for each sample and specific gene expression was evaluated using the 2ΔΔCT method. Gene expression levels were normalized to the glyceraldehyde 3-phosphate dehydrogenase GAPDH mRNA. Primers and probes for GAPDH (Hs02758991_g1), Osterix SP7 (Hs00541729_m1), runt-related transcription factor 2 Runx2/Cbfa1 (Hs00231692_m1), ALPI (Hs00357579_g1), and mitogen-activated protein kinase 8 MAPK8 (also known as JNK1, Hs01548508_m1) were all provided by Assays-on-Demand, Gene Expression Products (Applied Biosystems, Waltham, MA, USA).

### 2.10. Magnetized Hydrogel Preparation

Fibrin gel for cell embedding was prepared by mixing PBS-diluted fibrinogen and thrombin at the final concentration of 20 mg/mL and 5 U/mL, respectively. The cells were suspended in PBS (5 × 10^6^/mL) and quickly added to the mixture. Then, 10 µL of mixture were laden on to sterilized glass coated with hydro-repellant coating (SigmaCoat, Sigma-Aldrich, Schaffausen, Switzerland) to form a drop. The gel was then placed into the incubator (37 °C, 5% CO_2_) to allow the crosslinking to occur (in about 5 min).

### 2.11. Histological Staining

After in vitro culture, the 3D constructs were fixed overnight in a 4% paraformaldehyde solution, before being embedded into paraffin. Histological sections (4.5 µm thickness) were stained with Hematoxylin-Eosin (#GHS116 and #HT110116, respectively from Sigma-Aldrich, Schaffausen, Switzerland), and Alizarin Red S (#A5533, Sigma-Aldrich, Schaffausen, Switzerland). The primary antibody for anti-OCN was purchased from Abcam (ab198228, Cambridge, UK) and used at 1:500 dilution, before the sections were stained with the secondary antibody (1:300 dilution) coupled to Alexa488 (Life Technologies, Carlsbad, CA, USA) and 4′,6-diamidino-2-phenylindole (DAPI, Sigma-Aldrich, Schaffausen, Switzerland).

## 3. Results

### 3.1. Magneto-Sonoporation (MSP) Optimization

**Figure 1:**
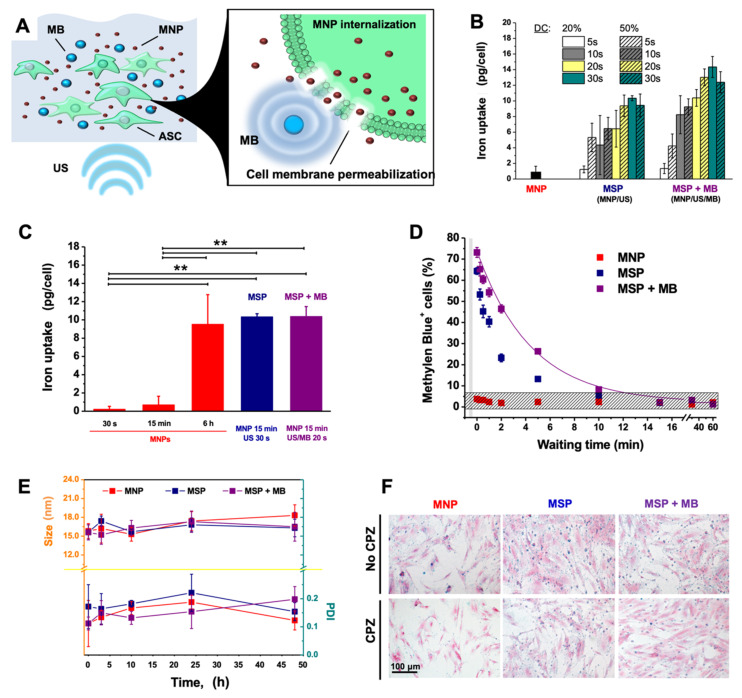
Iron uptake in sonoporated cells. (**A**) Diagram of the working principle of magneto-sonoporation (MSP) using ultrasound (US) in combination or not with microbubbles (MBs) to increase cell membrane permeability. (**B**) To optimize the sonoporation procedure, the cells underwent sonication with different duty cycles for different time ranges at 1.0 W/cm^2^. Other power intensities were also tested (data not shown). (**C**) The magnetic nanoparticles (MNP) uptake was compared among cells undergoing simple incubation for different time ranges (30 s, 15 min, 6 h), or upon sonication in the presence or absence of MBs (MSP and MSP + MB, respectively). ** *p* < 0:01. (**D**) In order to estimate the resealing time of the cell membrane after sonication, the cells were subjected to the Methylene Blue Permeability assay. Cells positive for the dye were considered to have altered membrane permeability and their fraction over the total population was plotted against time. (**E**) The stability of MNPs in culture media following MSP in the presence or absence of MBs was assessed by monitoring the mean hydrodynamic diameter (size, nm) and polydispersity index (PDI) via dynamic light scattering (DLS) over time, after dilution into cell culture media and maintenance in cell incubation (37 °C, 5% CO_2_ and pH 7.4). (**F**) Internalization of MNPs shown by Prussian Blue staining of ASCs after conventional incubation (6 h), MSP or MSP with MBs (15 min) in the presence or absence of the endocytosis-inhibitor chlorpromazine (CPZ). Equivalent volumes of phosphate-buffered saline (PBS) in the culture medium were used as controls (unlabeled).

In order to magnetize the ASCs, we perform MSP in the presence of a commercial formulation of IONPs, composed of pegylated magnetite nanoparticles with an average particle size of 15 nm. The sonoporation procedure was optimized across power intensity, exposure time, and duty cycle (i.e., exposure interval) in order select a combination of parameters that eventually led to high iron internalization rate with well-preserved cell viability. The intracellular uptake of IONPs, expected as a result of the membrane permeabilization (Figure 1A), was assessed by the spectrophotometric method. The highest iron uptake (≈14 pg/cell) was achieved in the cell group treated for 30 s with 20% duty cycle at 1.0 W/cm^2^ in the presence of MBs (Figure 1B). The iron content of cells incubated for 15 min with IONPs after 30 s sonication (10.35 pg/cell) was similar to that of cells exposed to US for only 20 s in the presence of MBs (10.4 pg/cell) (Figure 1C). IONPs accumulated in similar amounts also in the non-sonicated cells, but only after 6 h of incubation, whereas for short incubation times (30 s to 15 min) the internalization was minimal. The resealing time of the cell membrane after sonication was assessed by staining with the Methylene Blue, a dye that does not cross intact cell membranes and is employed as an indicator of altered membrane permeability (Figure 1D). During the recovery phase following the stimulation, the blue cells were counted at different time points. Their amount decreased from 73.1 ± 2.3% to 2.2 ± 0.2% in 15 min, revealing a decay constant of 4.5 min, which suggests a rapid return of the cell membrane to its physiological state. To assess whether the MNPs maintain the colloidal stability upon sonication, we used dynamic light scattering (DLS) to monitor the hydrodynamic diameter and polydispersity index (PDI) of particles diluted in the cell culture media and kept in cell culture conditions for two days (Figure 1E). The PDI values below 0.2 and the small variations in the particle diameter suggested a good stability of the suspension with no evidence of particle aggregation. Moreover, the MNPs internalized in the cells were detected by Prussian Blue staining (Figure 1F). The uptake of the nano-formulation mostly occurred via endocytosis, since the addition of the endocytosis-inhibitor chlorpromazine (CPZ) reduced the MNP internalization during conventional incubation, as shown by the weak staining. However, the endocytosis inhibition affected the internalization poorly when the cells were subjected to MSP.

### 3.2. Biological Effects of MSP

**Figure 2:**
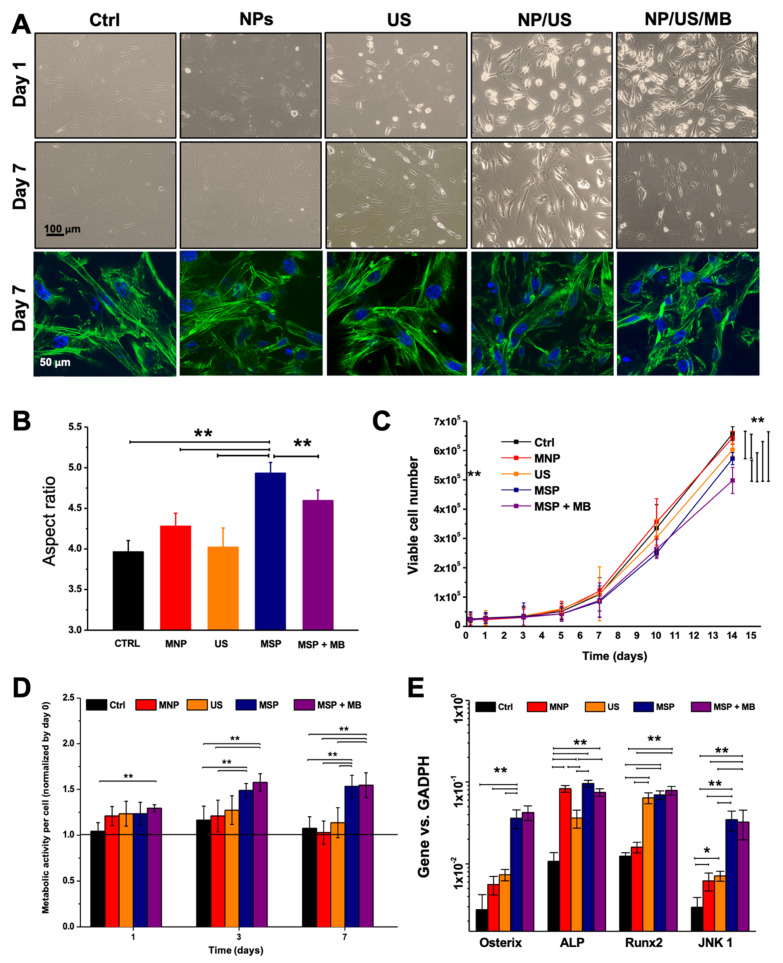
Biological effects. (**A**) Cell morphology was observed in phase contrast microscopy and in fluorescence microscopy (actin displayed in green, nuclei in blue). (**B**) Aspect ratio of cells was calculated one week after the various treatments. (**C**) Proliferation rate of ASCs measured as viable cell numbers over time. (**D**) Metabolic activity per cell in the different conditions, normalized to the DNA content, relative to day 0. (**E**) Gene expression of ASCs one week after sonoporation, focused on the expression of genes involved in osteogenesis and mechanical transduction. Asterisks indicate significant difference (by two-way analysis of variance (ANOVA)): * *p* < 0:05; ** *p* < 0:01. MNP = magnetic nanoparticles; US = ultrasounds; MB = microbubbles. MSP = magneto-sonoporation.

In order to understand if the MSP could affect the biology of ASCs, changes in their morphology were investigated. One day after MSP in the presence or absence of MBs, elongated cells and some rounded cells were observed by phase contrast microscopy (Figure 2A). Rounded cells were, however, mostly absent in sonicated cultures or in the untreated controls. Most of rounded cells were detached and most likely dead. One week after MSP, the cells displayed evident changes in the overall morphology with an augmented aspect ratio as compared to the other conditions (Figure 2A,B). Some cells with stellated morphology were also sporadically noticed (Figure 2A). Magneto-sonoporated cells also showed reduced proliferation ability as compared to the unsonicated ones (Figure 2C) and augmented metabolic activity with respect to all other controls (Figure 2D). Even if to a minor extent, their metabolic rate increased also in response to the sole presence of MNPs. Finally, the analysis of the gene expression profile revealed increased expression of osteogenesis marker genes (Osterix, ALP, Runx 2), and activation of a mechano-transduction signaling pathway (JNK1).

### 3.3. Ultrasound (US)-Activated 3D Matrices

**Figure 3:**
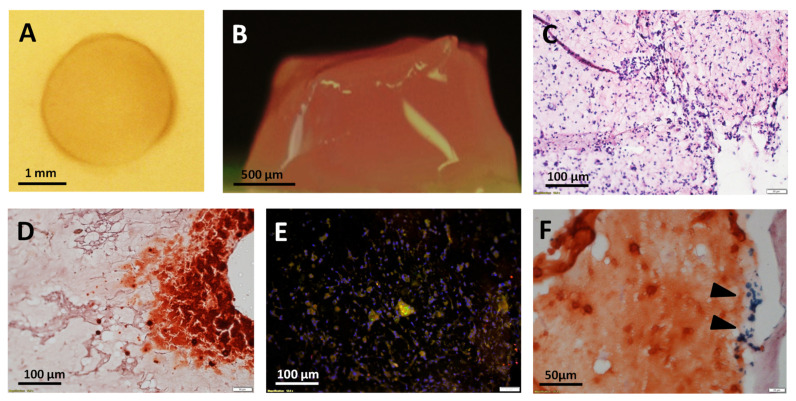
Sonoporation of magnetized 3D tissue constructs. A fibrin-based magnetized 3D tissue construct in top (in cell culture well) and lateral views ((**A**,**B**), respectively). Histological analysis of magnetized tissue, 7 days after sonoporation: hematoxilin and eosin (**C**), Alizarin Red (**D**) and osteocalcin (OCN) immunofluorescent staining (**E**). Nuclear staining (DAPI) and OCN staining are shown in blue and green, respectively. In Alizarin Red, clusters of MNPs were sporadically observed, as a result of aggregation (black arrows, (**F**)).

In order to understand whether certain biological effects mediated by MSP could be also observed into 3D cell culture environments, a hydrogel-based tissue construct was generated from the co-assembly of fibrin, MNPs and cells (Figure 3A,B), and then sonicated under the experimental conditions selected from the previous experiments. The histological analysis showed that, one week after treatment, the construct was densely populated by cells (Figure 3C), with some localized areas featuring calcium deposition and cells slightly positive for osteocalcin (OCN) expression, as shown by Alizarin Red staining and immuno-fluorescent staining, respectively (Figure 3D,E). Small clusters of aggregated MNPs were also visible in few sites of gels stained with Alizarin Red (Figure 3F).

## 4. Discussion

Biocompatible nanoscale materials have introduced outstanding innovation in biomedicine, finding use in the controlled delivery of biomolecules, manipulation of cell phenotype and behavior, and improvement of structural or biological features of substrates used for cell growth [50,51,52]. Key biological applications include non-invasive imaging and drug delivery in living organisms, as well as tissue engineering and wound healing [52,53,54,55,56,57,58]. In many instances, the nanomaterial internalization within cells is a mandatory step that can be achieved via various routes. In regards to IONPs, basic labeling procedures relying on the simple cell-nanoparticles co-incubation are conventionally applied because of their technical simplicity [10]. Some transfection agents, especially cationic compounds [8], can possibly be added, which include lipofectamine [9], poly-l-lysine [10,11] and protamine sulfate [12] among others. These substances coat the MRI contrast agents converting them from negatively to positively-charged. This facilitates the binding to the anionic cell membrane, and the subsequent cell internalization. However, such a process often takes a long time and requires high nanomaterial dosage that is detrimental when the exposure to the nano-formulation is associated with time- and dose-dependent decrease of cell viability [59]. The IONP-related cytotoxicity is often mediated by the production of reactive oxygen species [59,60]. In biological systems, the Haber–Weiss reaction is the major responsible mechanism for the generation of highly reactive hydroxyl radicals, and this process can be iron-catalyzed in response to exposure of ferric cores of IONPs let into unstable conditions (e.g., long-lasting permanence in biological milieu) [61,62,63]. In such conditions, the cells present gross structural alterations (for instance, at the level of the cytoskeleton) that eventually lead to functional impairment and also physical disruption [64]. In our study, we noticed a dramatic decrease in viability when the cells were incubated with IONPs not only together with lipofectamin, but also in presence of the positively-charged linear polymer polyethyleneimine (PEI) (data not shown) that is conventionally used for DNA transfection, but that also widely applied as IONP-coating agent [59]. This observation is in accordance with previous studies demonstrating that certain transfection agents hampered cell viability [59].

To overcome these issues, methods for instant labeling in the absence of additional chemicals have been given a lot of consideration [65]. In “magneto-electroporation” [13,14,15], electrical pulses induce electromechanical permeability changes in the cell membrane, eliciting the internalization of IONPs [14], or other metal-oxide based particles [15] with treatment duration in the time range of seconds. MSP also enables a fast cell magnetization by inducing a transient membrane perforation. In our experiments, we found that sonicating the cells for 30 s (or 20 s in the presence of MBs) led to IONP uptake yields that could be achieved only after 6 h of standard incubation. In contrast to magneto-electroporation, MSP relies on the convection of ultrasound waves originating from short electrical pulses [19,23]. The energy transfer involved is moderate [66], which implies safe technical settings that can be easily implemented into clinical applications. In the present study, substantial IONP internalization was achieved within a few seconds of sonication, but the treatment was not entirely inert to the cell biology. A short time after MSP, cell death was observed but only to a limited extent. However, even if dramatic cytotoxicity did not occur, sonoporated cells presented slightly decreased proliferation rates and increased metabolic activity. The alterations in the morphology and the genetic expression that we noticed in the treated cells might be the result of cell behavior modulation triggered by mechanical or chemical (iron-related) cues. This functional deviation is congruent to the potential evolution of the ASCs towards the osteogenic profile. In fact, we noticed that a morphology shift occurred in the magneto-sonoporated ASCs. In one week, they lost their conventional fibroblast-like morphology with flattened cell body and acquired instead more spindle-shaped or even stellate-shaped phenotypes, which are typical of the osteoblastic lineage. Congruently, the gene expression was altered by activation of genes involved in the osteogenic differentiation and mechano-transduction signaling pathways.

MBs are typical mediators of acoustic cavitation, a non-thermal US-induced bioeffect which is already widely used for intracellular-delivery [18,19,20,67]. We performed some of the experiments in the presence of MBs to understand whether they render the MSP more efficient and can modulate the cell behavior. It was found that the presence of MBs could influence some of the cellular processes under investigation (such as the proliferation and the internalization of MNPs), but only scarcely affected others (like the gene expression). This suggests that they play a role in the MNP-cell interaction and can affect the cellular behavior but only to a limited extent.

Methods for bio-physical cell stimulation have started to be integrated into 3D cell culture models, in order to explore their applicability into systems that more faithfully resemble the native tissue configuration as compared to cell monolayers grown in plastic dishes. Matrices activated by sonoporation have also been presented [68,69,70,71,72], which were intended to augment the cell internalization of genetic material on demand. In our study, we have obtained a matrix for ASC culture by co-assembling the MNPs and a common biomedical hydrogel. Our preliminary data show that such culture material can mediate acoustic stimulation of cell functions. The biological effects that we observed also included the deposition of calcium, a feature that points to an osteogenic switch of the ASC phenotype. It is worth noting that the previously reported sono-activated matrices were also developed to enhance the osteogenic potential of progenitors but they relied on direct genetic cell modification [70,71,72], while in our work we demonstrated that similar results can be achieved by using the mechanical action conveyed by applied forces and small scaled materials. However, the main limitation of the present study is that the biomolecular mechanisms underlying the observed biological effects are not well elucidated. In particular, deeper investigations are needed to differentiate the biochemical effects of the sole MNPs from those of the mechanical stress induced by the MSP procedure. To such a purpose, future studies should focus on the comparison of the MSP with conventional incubation conditions that allow to internalize similar amounts of IONPs, as well as on the assessment of variations in the iron metabolism. Moreover, a complete characterization of the specific formulation of MNPs used as sonoporation agents is also necessary in order to identify the relation between the chemo-physical properties of the nanomaterials, the applied forces, and the reaction triggered in the stimulated biological system. Nevertheless, although observational, the results here reported reveal clear biological alterations that might impact on the proper use of the cells and represent a novel method for biophysical modulation of cell functions.

In the past research, MSP was demonstrated in a few types of stem cell, some of which are endowed with limited potency and can treat only specific pathological tissues [22,23,32]. For instance, it was shown that neural stem cells labeled with superparamagnetic IONPs through focused US maintained key biological features, such as differentiation potential and migration ability to the diseased tissue sites [22,23]. In our study, we performed MSP on ASCs, stem cells characterized by accessible extraction sites, multipotency, and simple culture, that can serve in the regenerative therapy of various tissue types [33,34,35,36,37,38,39]. However, we discovered morphological and biochemical alterations suggesting that magneto-sonoporated ASCs were committed towards the osteoblastic profile, thus highlighting a more precise applicative direction in the bone tissue repair.

As the MSP technical settings herein tested allowed for biosafe treatment of cells in vitro, the future pre-clinical research will benefit from our technical optimization of the procedure, while focusing on the mechanisms causing the observed biological alterations. We performed our experiments in a closed apparatus where focused ultrasounds were delivered to cell suspensions collected in plastic tubes, as described in previous works [22,23]. In such a setting, a large number of cells can be easily and safely labeled immediately after the potential extraction from patients, with a reduced contamination risk. The US emission powers were included in the typical value ranges for biocompatible sonoporation, already employed in previous works where MSP demonstrated high capability to preserve cell viability and integrity [22,23,30,31,32]. The translatability of the present MSP setting mostly concerns the possibility to enhance and accelerate the cell magnetization process, and to control the cell functional profile in vitro, before the potential re-implantation of cells in vivo. Nevertheless, a direct use of MSP in patients can be prospectively imagined following crucial achievements that include:

i. a better definition of the mechanobiological activity of sonicated MNPs and the mechanisms triggering the biological alterations observed in the cells;
ii. the influence of the biophysical properties of the specific MNP formulation on the MSP process;
iii. the understanding of the interaction of US-mediated physical forces with human tissues and bodies;
iv. the inherent optimization of the sonication setting.

The technical implementation of such a knowledge might render it possible to design direct applications of MSP in vivo or to engineer implantable sonoactivated matrices. In this regard, our preliminary test on 3D hydrogel matrices might represent the basis for future investigations enabling the transition of the MSP from a cell pre-conditioning technique to an imaging and therapeutic method applicable in vivo.

## 5. Conclusions

As a rapid and convenient technique for stem cell labeling, MSP has good prospects for clinical investigations, and the optimization of parameters and experimental settings will allow the MNP-cell interactions to be ameliorated [22]. However, an important consideration arising from our study is that the safety of MSP as applied to stem cell imaging and therapy results in being challenging to decipher and must be evaluated by distinguishing the effects occurring at two different levels. On the one hand, the US waves can have high mechanical compliance with biological materials, rendering the MSP relatively safe to use with cells. Therefore, its application to biological systems is sustainable, and preserves their integrity and survival. On the other hand, the impact of the biophysical stimulation on the intracellular processes has to be carefully assessed. As demonstrated in this work, US and/or micro/nano-materials can strongly affect the cell phenotype in both monolayer and tissue-like configurations. Despite raising questions in regard to the labeling of transplantable cells, this aspect also opens intriguing perspectives in strategies for remote control over cell functions.

## Data Availability

Data sharing is not applicable.
